# A novel DNA repair‐related nomogram predicts survival in low‐grade gliomas

**DOI:** 10.1111/cns.13464

**Published:** 2020-10-16

**Authors:** Guanzhang Li, Fan Wu, Fan Zeng, You Zhai, Yuemei Feng, Yuanhao Chang, Di Wang, Tao Jiang, Wei Zhang

**Affiliations:** ^1^ Department of Molecular Neuropathology Beijing Neurosurgical Institute Capital Medical University Beijing China; ^2^ Department of Neurosurgery Beijing Tiantan Hospital Capital Medical University Beijing China; ^3^ Center of Brain Tumor Beijing Institute for Brain Disorders Beijing China; ^4^ China National Clinical Research Center for Neurological Diseases Beijing China; ^5^ Chinese Glioma Genome Atlas Network (CGGA) Asian Glioma Genome Atlas Network (AGGA)

**Keywords:** DNA repair functions, low‐grade glioma, prognosis prediction, recurrence prediction, tumor recurrence

## Abstract

**Aims:**

We aimed to create a tumor recurrent‐based prediction model to predict recurrence and survival in patients with low‐grade glioma.

**Methods:**

This study enrolled 291 patients (188 in the training group and 103 in the validation group) with clinicopathological information and transcriptome sequencing data. LASSO‐COX algorithm was applied to shrink predictive factor size and build a predictive recurrent signature. GO, KEGG, and GSVA analyses were performed for function annotations of the recurrent signature. The calibration curves and C‐Index were assessed to evaluate the nomogram's performance.

**Results:**

This study found that DNA repair functions of tumor cells were significantly enriched in recurrent low‐grade gliomas. A predictive recurrent signature, built by the LASSO‐COX algorithm, was significantly associated with overall survival and progression‐free survival in low‐grade gliomas. Moreover, function annotations analysis of the predictive recurrent signature exhibited that the signature was associated with DNA repair functions. The nomogram, combining the predictive recurrent signature and clinical prognostic predictors, showed powerful prognostic ability in the training and validation groups.

**Conclusion:**

An individualized prediction model was created to predict 1‐, 2‐, 3‐, 5‐, and 10‐year survival and recurrent rate of patients with low‐grade glioma, which may serve as a potential tool to guide postoperative individualized care.

## Graphical abstract

Based on large sample of clinical and sequencing data, we constructed an individualized prediction model for recurrence time predicting. The prediction model is accurate and simple enough to be widely applied for postoperative individualized care guidance of low‐grade gliomas.

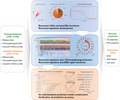

## 1 INTRODUCTION

Diffuse low‐grade gliomas (LGGs) are infiltrative, incurable lesions characterized by a continuous slow‐growth and an almost unavoidable anaplastic transformation.^1^, ^2^, ^3^ Median overall survival for patients with LGGs ranges from 5.6 to 13.3 years depending on tumor histopathologic feature, molecular phenotype, and growth rate.^2^, ^4^, ^5^ Unlike their high‐grade glioma counterparts, low‐grade glioma with a more favorable prognosis pose unique challenges for both clinicians and patients for time‐consuming monitoring of tumor recurrence.^6^ An individualized plan of postoperative imaging assessment will facilitate the efficient use of medical resources and reduce medical costs.

Postoperative individualized care plan based on the highly accurate individualized recurrence prediction model. Nomograms, presenting the results of predictive models in a printed format, could be widely used in clinical practice.^7^ Numerous nomograms for Overall (OS) and Progression‐Free Survival (PFS) prediction have been built in patients with neuroendocrine tumors,^8^ renal cell carcinoma,^9^ nasopharyngeal carcinoma,^10^ and oropharyngeal cancer.^11^ However, the relative rarity of LGG combined with long overall survival has hindered the construction of a high‐accuracy predictive model. The Chinese Glioma Genome Atlas (CGGA) project includes the clinical, sequencing, and long‐term follow‐up data of primary and recurrent low‐grade gliomas with the largest sample size, providing the possibility for genetic analysis and prediction model construction of recurrent LGGs.

Incomplete tumor resection and treatment resistance are important reasons for LGGs recurrence.^12^, ^13^ The proliferation capacity of the remaining tumor cells determines the time of tumor recurrence. DNA repair ability of tumor cells determines the sensitivity of patients to postoperative adjuvant therapy. Since DNA repair processes and cell cycle processes have some similarities in biological functions, we believed that both of them play an important role in tumor recurrence. Therefore, the prediction model based on the above functions can be applied to the PFS prediction of LGGs.

The objective of this study was to explore the biological features of recurrent LGGs and create a prognostic model, which incorporates genome features and clinical risk factors that can accurately predict the recurrence probability as different time points. This study provided a treatment‐guidance tool for individualized postoperative care of LGGs.

## 2 METHODS

### 2.1 Material and Methods

In total, 188 patients with primary or recurrent LGG were enrolled in the training group and 103 patients in the validation group. Resected tumor samples were immediately placed in liquid nitrogen and only samples with more than 80% tumor cells, judged by HE staining of adjacent tissues, were selected for further sequencing. Transcriptome data of LGG samples were generated by the Agilent platform. Molecular testing was performed at the Molecular Pathology Testing Center of Beijing Neurosurgical Institute. All patients were followed up trimonthly by telephone or clinic for an average of 1813 days. 15 of 188 patients (7.98%) lost to follow‐up in the training group and 5 of 103 patients (4.85%) lost to follow‐up in the validation group. Clinical information of patients was summarized in Table S1.

The sequencing data, clinical, and follow‐up information of primary and recurrent LGG patients were uploaded to the CGGA portal (http://cgga.org.cn/). All datasets used and/or analyzed in this study are available from the corresponding author on reasonable request.

### 2.2 Biological functional enrichment scores

The biological functional enrichment score of each patient was generated by Gene Set Variation Analysis (GSVA) analysis based on tumor transcriptome sequencing data. GSVA analysis was performed using the default parameters by the *gsva* package in R as described in the previous study.^14^ Gene list for each biological function was downloaded from AmiGO 2 Web portals (http://amigo.geneontology.org) most recently.

### 2.3 LASSO‐COX dimension reduction analysis

LASSO‐COX dimension reduction analysis was performed by *glmnet* and *survival* packages in R. The λ value corresponding to the minimum partial likelihood deviance was selected as the optimal λ in our study. Finally, 4 candidate genes and corresponding lambda values (CBX8: 0.136440456719623, EYA1: −0.0196495722505206, FOXM1: 0.0258928486777096, and H2AFX: 0.00175903850740734) were obtained based on PFS of LGG patients in the training group. The recurrent score of each patient was calculated as follows:

Recurrent score = expr_CBX8_ × λ_CBX8_ + expr_EYA1_ × λ_EYA1_ + expr_FOXM1_ × λ_FOXM1_ + expr_H2AFX_ × λ_H2AFX_.

where expr_gene_ was the expression level of the gene and λgene was the corresponding lambda value.

### 2.4 Nomogram construction

Nomogram analysis was constructed in the training group by *rms* package in R. The upper part is the scoring system and the lower part is the prediction system. The 1‐, 2‐, 3‐, 5‐, and 10‐year survival and recurrent rate of LGG patients could exactly be predicted by total points, sum points of every factor. Verification of the prediction accuracy of OS and PFS was performed in patients of the validation group. Calibrate curves and C‐Index values were used to show the accuracy of the survival prediction.

### 2.5 Statistical analysis

Statistical analyses were executed using R (https://www.r-project.org/, v3.5.0), SPSS software (IBM, v25.0, Chicago, IL), and GraphPad Prism (v8.0, La Jolla, CA). The prognostic value was evaluated by Kaplan‐Meier analysis and COX analysis. GSEA analyses were implemented with GSEA package in java software (http://software.broadinstitute.org/gsea/index.jsp) and gene ontology (GO) was performed in the DAVID portal website (https://david.ncifcrf.gov/summary.jsp). For all statistical methods, *P* < 0.05 was considered as significant difference.

## 3 RESULTS

### 3.1 DNA repair functions significantly enriched in recurrent low‐grade gliomas

**Figure 1:**
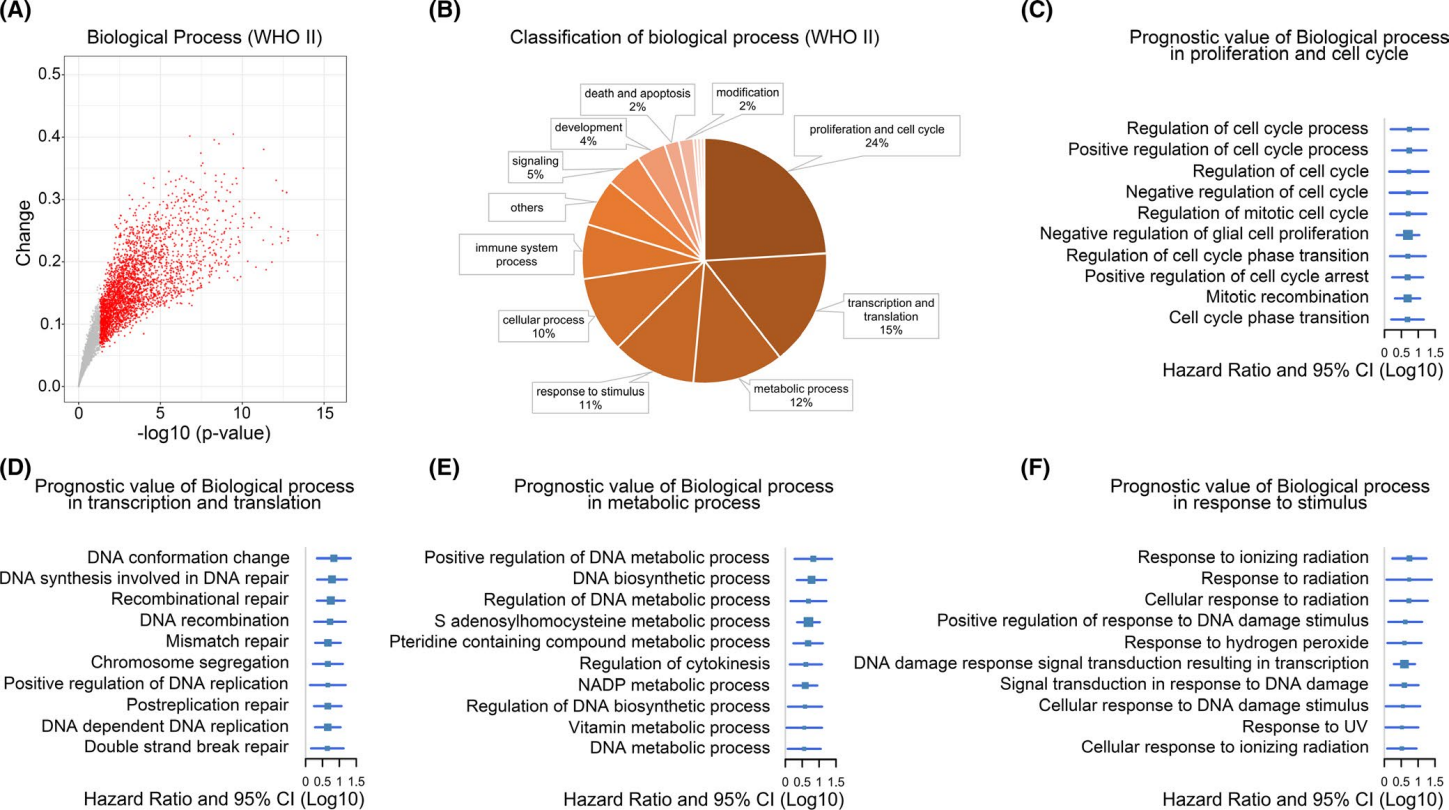
The landscape of highly activated biological processes in recurrent low‐grade gliomas. A, Highly activated biological processes in recurrent LGGs compared to primary tumors. Red dots were significantly elevated BPs. Gray dots represented the non‐significant changed BPs. B, Classification of the recurrent LGGs enriched BPs. Number of BPs in a certain group divided by the total number of significantly changed BPs to get the percentage of each group. C, BPs with the most prognostic value in proliferation and cell cycle group. D, BPs with the most prognostic value in the transcription group. E, BPs with the most prognostic value in the metabolic process group. F, BPs with the most prognostic value in response to the stimulus group

A 5917 biological functional enrichment scores for 138 primary and 50 recurrent low‐grade glioma patients were calculated by the GSVA algorithm. We found that 2596 biological functions were significantly increased in recurrent tumors, while 108 biological functions were significantly decreased (Figure 1A). Classification of significantly elevated biological functions in recurrent tumors found that proliferation and cell cycle (24%), transcription and translation (15%), metabolic process (12%), and response to stimulus (11%) account for the highest proportion (Figure 1B). The biological functions related to tumor progression‐free survival (PFS) in low‐grade glioma were screened out by multivariate COX analysis. The biological functions most related to PFS in each classification were shown in Figure 1C‐F. As expected, the results suggested that faster cell cycle, increased DNA repair and biosynthesis, and cellular response to radiation were significantly elevated in recurrent tumors.

### 3.2 Development of a recurrent signature for low‐grade gliomas

**Figure 2:**
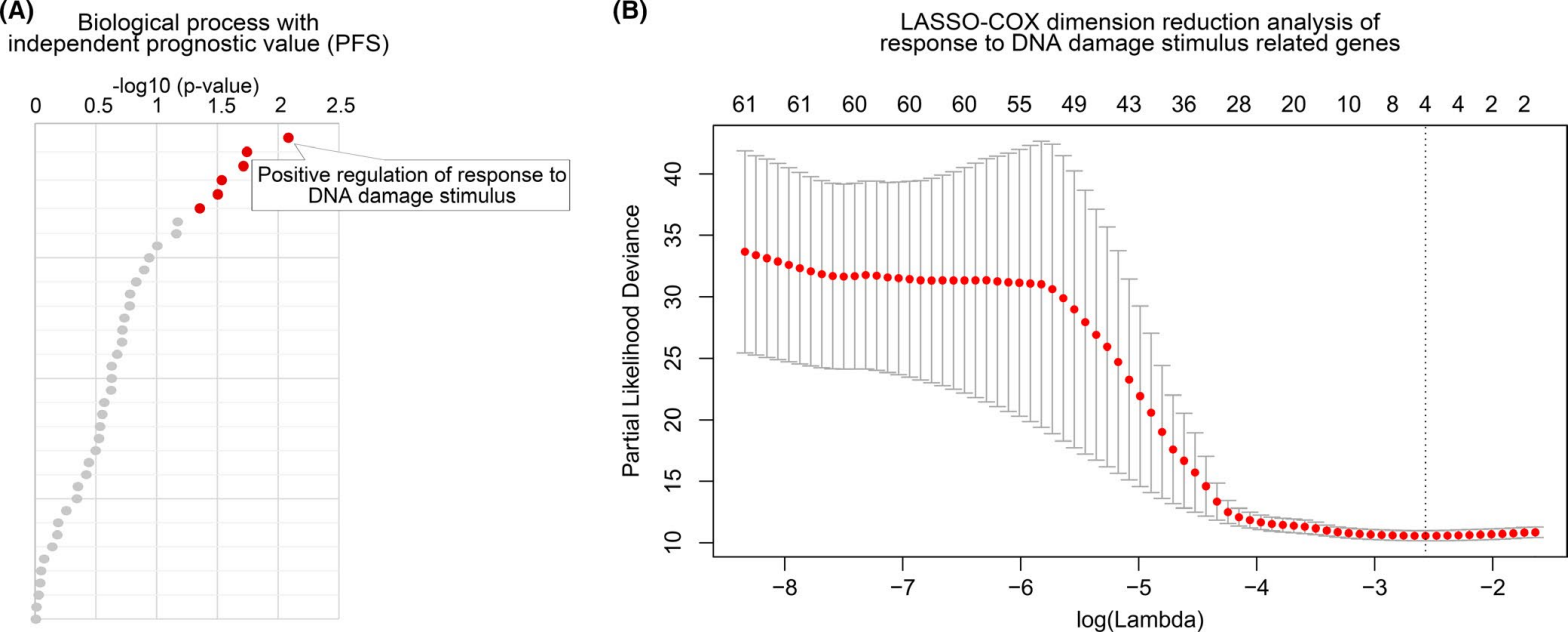
Building a recurrent signature by LASSO‐COX analysis. A, BPs with independent prognostic value in LGGs. Red dots were BPs independent prognostic value. BPs stained gray was not an independent prognostic factor. B, Screening the most representative 4 genes in response to DNA damage stimulus‐related genes by LASSO‐COX analysis

The biological functions related to PFS were included in multivariate COX analysis to screen independent prognostic functions. Positive regulation of response to DNA damage stimulus was screened out as a function that was significantly elevated in recurrent gliomas and had the most independent prognostic value for PFS (Figure 2A). Subsequently, a recurrent signature based on positive regulation of response to DNA damage stimulus‐related genes was constructed by LASSO‐COX dimension reduction analysis (Figure 2B). Finally, four candidate genes (CBX8, EYA1, FOXM1, and H2AFX) and their corresponding lambda values were used to calculate the recurrence score for each patient. The median recurrence score (0.566) of the training database was set as the cutoff value.

### 3.3 The recurrent signature can stably predict the prognosis of patients with low‐grade glioma

**Figure 3:**
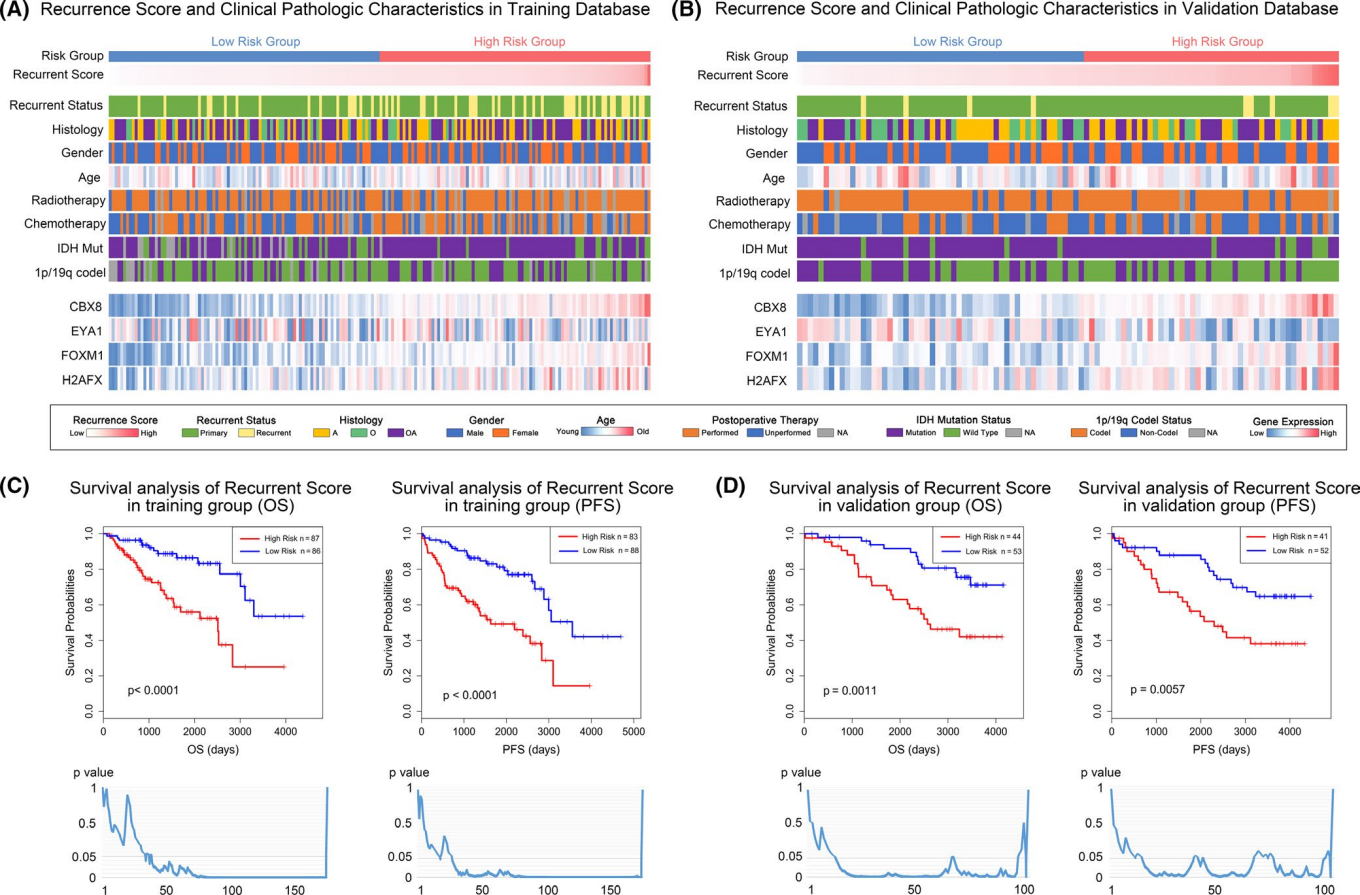
The relationship between the recurrent score and clinical characteristics and survival in patients with LGGs. (A and B) The heatmap showed the clinical‐pathologic factors and 4 representative genes for each LGG in ascending order of the recurrence score in training and validation groups. C, The Kaplan‐Meier curves indicated that patients in high‐risk group have shorter OS and PFS than patients in the low‐risk group. The line chart showed the *P* values of survival analysis between patients with lower and higher recurrence scores with various cutoff. D, The recurrence score showed good predictive accuracy in the validation group

Patients in different recurrence risk groups showed distinct patterns of clinical‐pathologic characteristics. In the training database, tumor recurrent status and IDH mutation status showed asymmetric distribution characteristics with the increase of recurrent score (Figure 3A). In the validation database, tumor recurrent status, IDH mutation status, and 1p/19q codeletion status showed distinct patterns in different recurrent risk groups (Figure 3B). The recurrent signature showed superior predictive values for overall survival and PFS in both training and validation databases (Figure 3C,D). Importantly, the recurrent signature showed prognostic significance in low‐grade gliomas even with different arbitrary risk value cutoffs. Univariate and multivariate COX analysis revealed that the recurrent score was an independent prognostic factor in the training and validation database (Table [Link], [Link], [Link], [Link]). The ROC curve was performed to verify the accuracy of the recurrent score in prognostic prediction (Figure S1A‐D). Also, the predictive role of the recurrent score was further verified in other LGG databases (Figure S1E,F).

### 3.4 Relationship between recurrent scores of the recurrent signature and clinical‐pathologic characteristics

**Figure 4:**
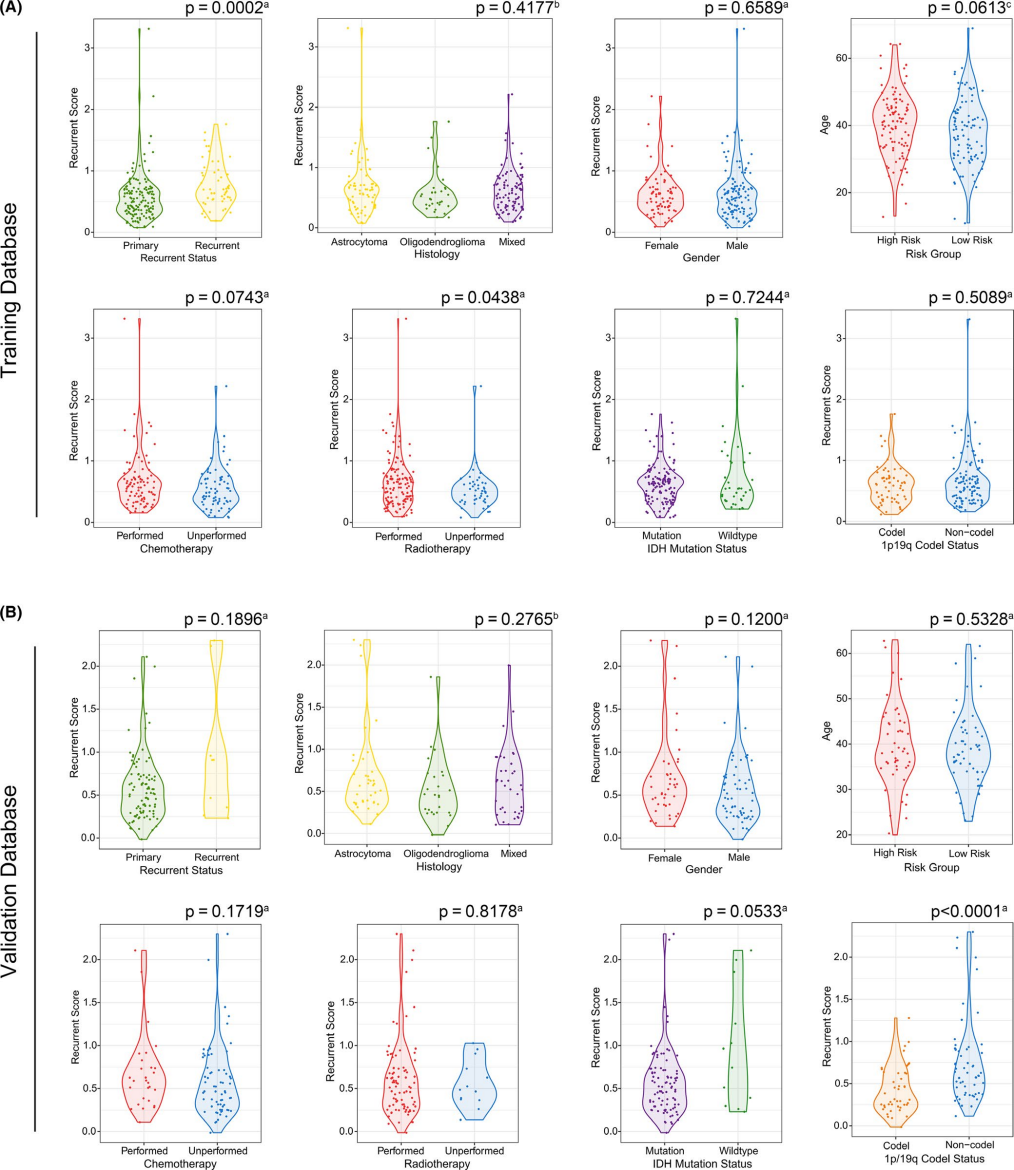
The relationship between recurrent scores and clinical‐pathologic characteristics of LGGs. (A and B) Violin charts showed the distribution of recurrent scores between different clinical‐pathologic characteristics of LGGs in training and validation groups. The significance of the difference between the two groups was verified by ^a^ *Mann Whitney test* or ^c^ *Student's* t*‐test*. The significance of the difference between the three groups was verified by ^b^ *Kruskal‐Wallis test*

The relationship between recurrent scores and clinical‐pathologic factors was further tested. The recurrent score significantly increased in recurrent tumors and moderately slightly increased in patients with postoperative radiotherapy in the training database (Figure 4A). The recurrent score significantly increased in 1p/19q non‐codeletion tumors in the validation database (Figure 4B). However, the recurrent score showed no correlation with histology, gender, age, postoperative chemotherapy, and IDH mutation status in both training and validation databases.

### 3.5 The recurrent score is closely related to cell division and DNA metabolism

**Figure 5:**
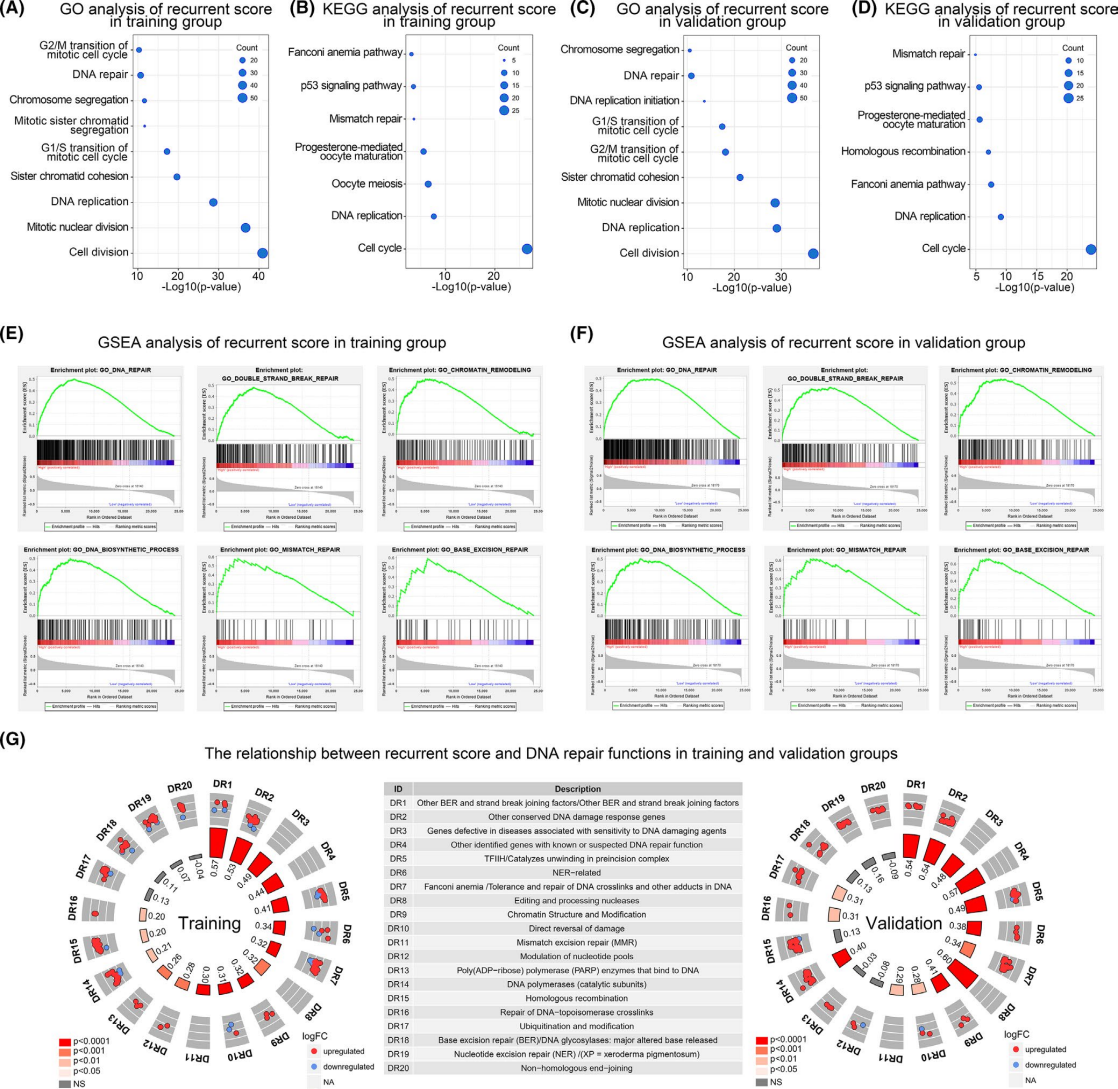
Biological functions associated with the recurrent scores. (A and B) The recurrent score related biological process and pathways revealed by Gene ontology analysis and KEGG analysis in the training group. (C and D) The recurrent score related biological process and pathways revealed by Gene ontology analysis and KEGG analysis in the validation group. (E and F) GSEA analysis showed that the recurrent score was closely related to DNA damage repair functions. G, The recurrent score was significantly positively correlated with most DNA repair‐related functions. The R‐value of *pearson* correlation analysis of recurrent scores and DNA repair functions enrichment scores were showed in the inner circle. The strength of the correlation was represented by the shade of red

To explore the biological functions and pathways associated with the recurrent score, the Gene Ontology (GO) and Kyoto Encyclopedia of Genes and Genomes (KEGG) functional enrichment analysis and Gene Set Enrichment Analysis (GSEA) were performed. After screening the genes most related to the recurrent score, GO and KEGG analyses were performed based on these genes. GO analysis showed that the recurrent score was closely related to G2/M transition of mitotic cell cycle and DNA repair in training and validation databases (Figure 5A,C). KEGG analysis showed that the recurrent score was closely related to the p53 signaling pathway and mismatch repair in both databases (Figure 5B,D). The close relationship between the recurrent score and DNA repair‐related functions was further verified by GSEA analysis in training and validation databases (Figure 5E,F).

### 3.6 The recurrent score is closely related to the DNA repair function

The relationship between the recurrent score and DNA repair function was further explored. Functional enrichment scores of DNA repair functions of each patient were calculated. Correlation analysis found that the recurrent score was significantly positively correlated with most DNA repair functions. The recurrent score was significantly positively correlated with 16 DNA repair functions in the training database. In the validation database, the recurrent score was significantly positively correlated with 14 kinds of DNA repair functions (Figure 5G).

### 3.7 The individualized prediction model showed robust predictive accuracy

**Figure 6:**
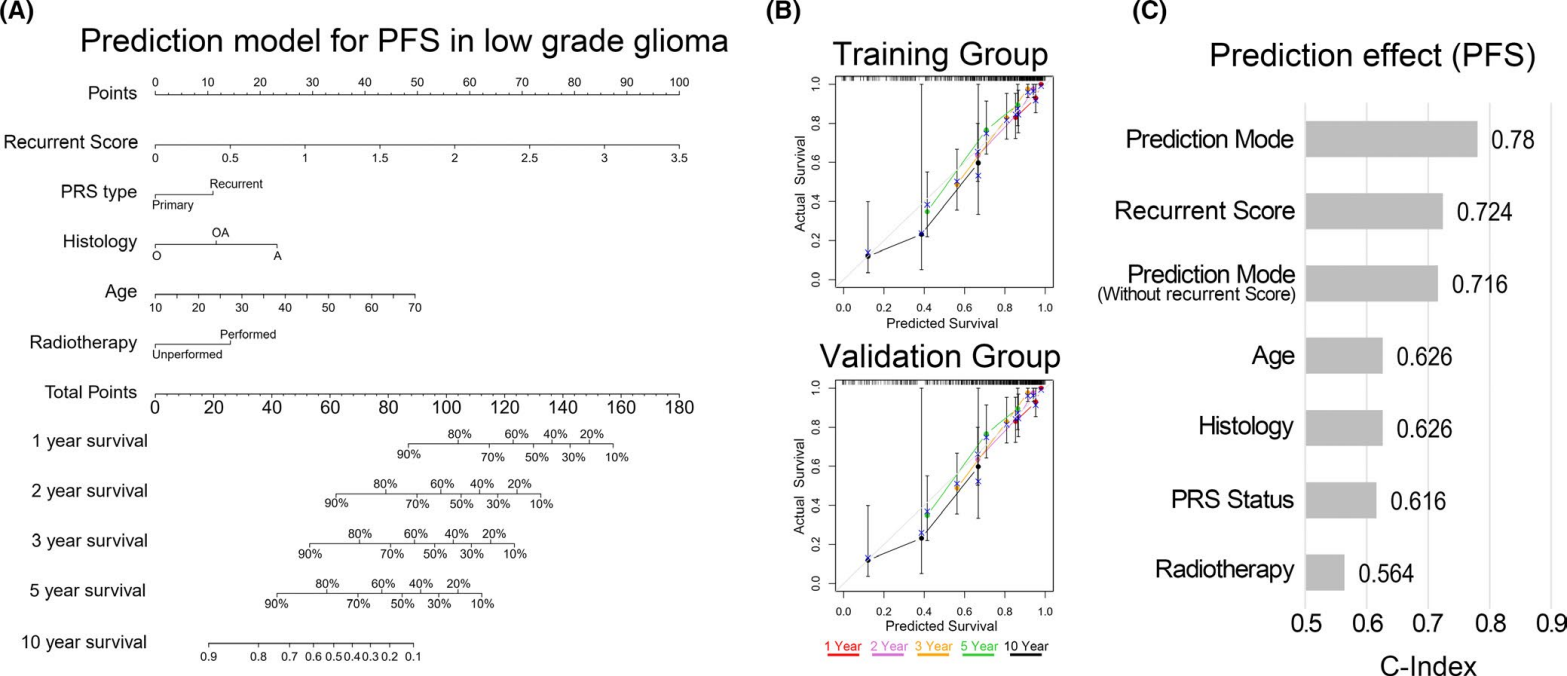
The individualized prediction models for PFS in LGGs. A, The 1‐, 2‐ 3‐, 5‐, and 10‐year recurrent rate of LGG patients after tumor resection could exactly be predicted by the nomogram. B, The Calibration plots showed the comparison between predicted and actual PFS for 1‐, 2‐ 3‐, 5‐, and 10‐year survival probabilities in training and validation groups. C, The predictive effect of the individualized prediction model, recurrent score, prediction model without the recurrent score, and clinical prognostic factors of LGGs on PFS was evaluated by C‐Index

To facilitate the clinical application of the prognostic prediction model, an individualized prediction model was constructed. The individualized prediction model for PFS prediction was constructed based on the independent predictive factors, including recurrent score, primary/recurrent status, histology, age, and postoperative radiotherapy. Figure 6A showed that the (1‐,2‐, 3‐,5‐ and 10‐year) tumor recurrence probability of low‐grade glioma patients could be estimated by the individualized prediction model. The nomogram and actual observations in the calibration curve showed a satisfactory overlap in training and validation databases, indicating an optimal predictive accuracy (Figure 6B). The C‐index of this nomogram model was 0.78, which is higher than any other prediction model (Figure 6C). To further expand the application range of our prediction model, the individualized prediction model for OS prediction based on predictive factors, including recurrent score, primary/recurrent status, histology, age, postoperative radiotherapy, and IDH mutation status was also established for (1‐,2‐, 3‐,5‐ and 10‐year) survival probability prediction of low‐grade glioma patients (Figure S2A). The OS prediction model also showed robust predictive accuracy (Figure S2B,C).

## 4 DISCUSSION

Monitoring of early recurrence in postoperative low‐grade glioma patients is important for clinical practice. Although some recurrence‐predicting studies in glioma have been performed, few reports have been able to predict the recurrence or prognosis of low‐grade glioma patients accurately due to insufficient samples with long‐term follow‐up.^12^, ^15^, ^16^, ^17^, ^18^, ^19^ If an individualized prediction model predicting early recurrence could be achieved with high accuracy, it would be possible to make better clinical decisions, which might improve a patients’ prognosis.

Taking the advantages of the CGGA database, prediction models for the recurrence or prognosis prediction were developed and validated based on low‐grade glioma patients with long‐term follow‐up (Training: up to 4374 days and Validation: up to 4163 days). Importantly, transcriptome sequencing data of tumor tissue from these patients were also available for analysis. Among 5917 biological functions, positive regulation of response to DNA damage stimulus was screened out as a significantly elevated function in recurrent tumors. The LASSO‐COX dimension reduction analysis was used to select an optimal prognostic signature with the most representative gene markers for the identification of the 4‐gene signature in recurrent low‐grade gliomas. Then, a novel recurrent score based nomogram was constructed to predict early recurrence in patients with LGG following curative resection. The nomogram, incorporating the Recurrent score, P/R status, Histology, Age, IDH1 Status, and Radiotherapy Status, successfully identify patients at high risk of early recurrence. Some studies have reported that the extent of tumor resection and tumor location are important prognostic factors for LGG.^13^, ^20^, ^21^ We further explored the relationship between recurrent score and tumor location. As shown in Figure S3, there is no significant correlation between the recurrent score and tumor location in both training and validation databases. Furthermore, the nomogram provided better predictive accuracy than the clinical factor‐based model or recurrent signature alone, demonstrating the incremental value of the nomogram to the current early diagnosis of recurrent LGG. Moreover, our nomogram is easy to use, and it could serve as a quick and efficient tool for individualized prediction of prognosis and for guiding treatment in recurrent LGG patients.

As a standard adjuvant treatment for low‐grade gliomas, postoperative radiotherapy and chemotherapy kill tumor cells by inducing DNA damage.^4^, ^6^ The unrepaired DNA damage is also a major source of potentially mutagenic lesions that promotes malignant progression of tumor.^22^, ^23^ Therefore, response to DNA damage is a key factor in tumor progression and recurrence of LGG. Our study found that response to DNA damage is a key factor in the recurrence of LGGs, and that DNA damage response‐based signature is independent of the clinicopathological state of patients, such as histology, gender, WHO risk status, and postoperative treatment.^5^, ^24^ Further analysis confirmed that the recurrent score, as an accurate reflector of the DNA repair function, was constructed as a robust predictor of recurrence of LGG. This study suggested that DNA repair function targeted therapy may prevent the progression and recurrence of LGG. The recurrent score can also be used as a predictor of the sensitivity of targeted therapy.

As a clinical application tool, our nomogram included only routine clinical examination items for glioma and did not use factors that may require statistical software or trained analysts such as tumor volume, the extent of resection, and epilepsy seizure types.^25^, ^26^ Although not perfect, this represents an encouraging level of predictive accuracy. Calibration shows how closely the predicted probabilities agree numerically with the actual outcomes. Of note, easily acquired factors and user‐friendly operation methods make this prediction model more widely applicable. An online individualized prediction model is being developed. Clinicians without special training, or even patients themselves, will be able to predict tumor survival and recurrence through online operations in the near future.

The present study contains several limitations. A limited sample size may affect the model training. With the widespread application of the individualized prediction model, the parameters and predictive factors of the model may need to be updated to achieve higher prediction accuracy. The limited sample size also leads to the deviation of results in the relationship between the recurrence score and clinicopathological characteristics. Besides, the calculation of recurrent score requires a test kit, which increases the workload of pathologists as well as the cost of patients. Therefore, the test kits need to be more convenient and cheaper or replaced by other methods, such as radiomics. But it is worth noting that convenience and cost are contradicted, and we need to constantly explore the best balance in clinical applications.

In conclusion, the current research not only provides a tool for the objective assessment of the recurrence probability and survival rate of postoperative LGG, but also provides a theoretical basis for the targeted therapy of recurrent LGG. The individualized prediction model is simple and accurate enough to be widely applied to a broad clinical setting.

## CONFLICT OF INTEREST

The authors declare no potential conflicts of interest.

## ETHICAL APPROVAL

This study was approved by Capital Medical University Institutional Review Board (IRB). Written informed consents were obtained from the patients (or their families) for the CGGA project.

## Supporting information

Fig S1

Fig S2

Fig S3

Table S1

Table S2

Table S3

Table S4

Table S5

## Data Availability

The sequencing data, clinical, and follow‐up information of primary and recurrent LGG patients were uploaded to the CGGA portal (http://cgga.org.cn/). All datasets used and/or analyzed in this study are available from the corresponding author on reasonable request.
