# Supplementary figures and images for: A novel DNA repair‐related nomogram predicts survival in low‐grade gliomas
Source: CNS Neurosci Ther. 2020 Oct 16;27(2):186–95. doi: 10.1111/cns.13464 (PMC7816205; doi:10.1111/cns.13464)

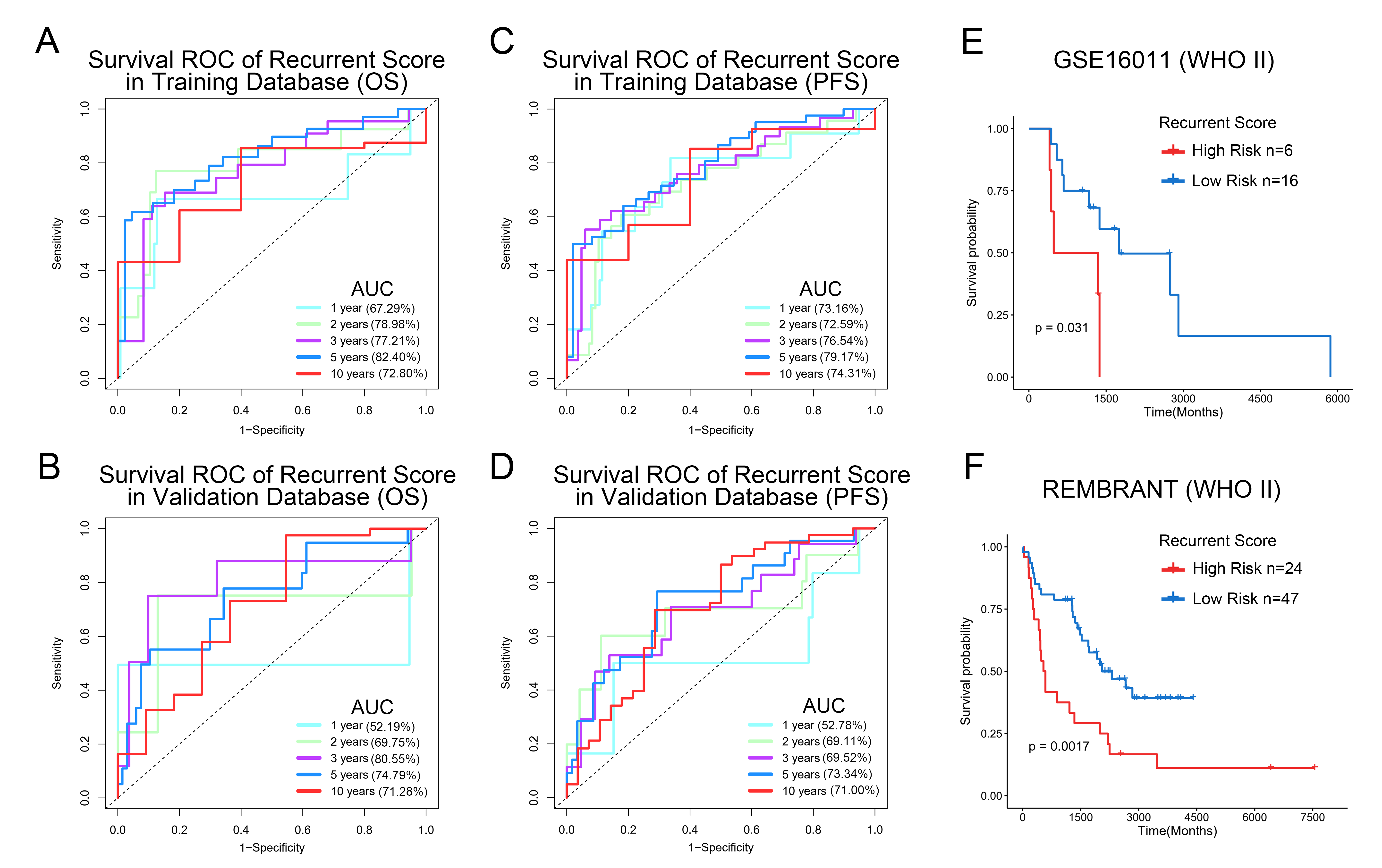

Supplement: Supplementary file 1 — Fig S1 [file CNS-27-186-s001.tif]

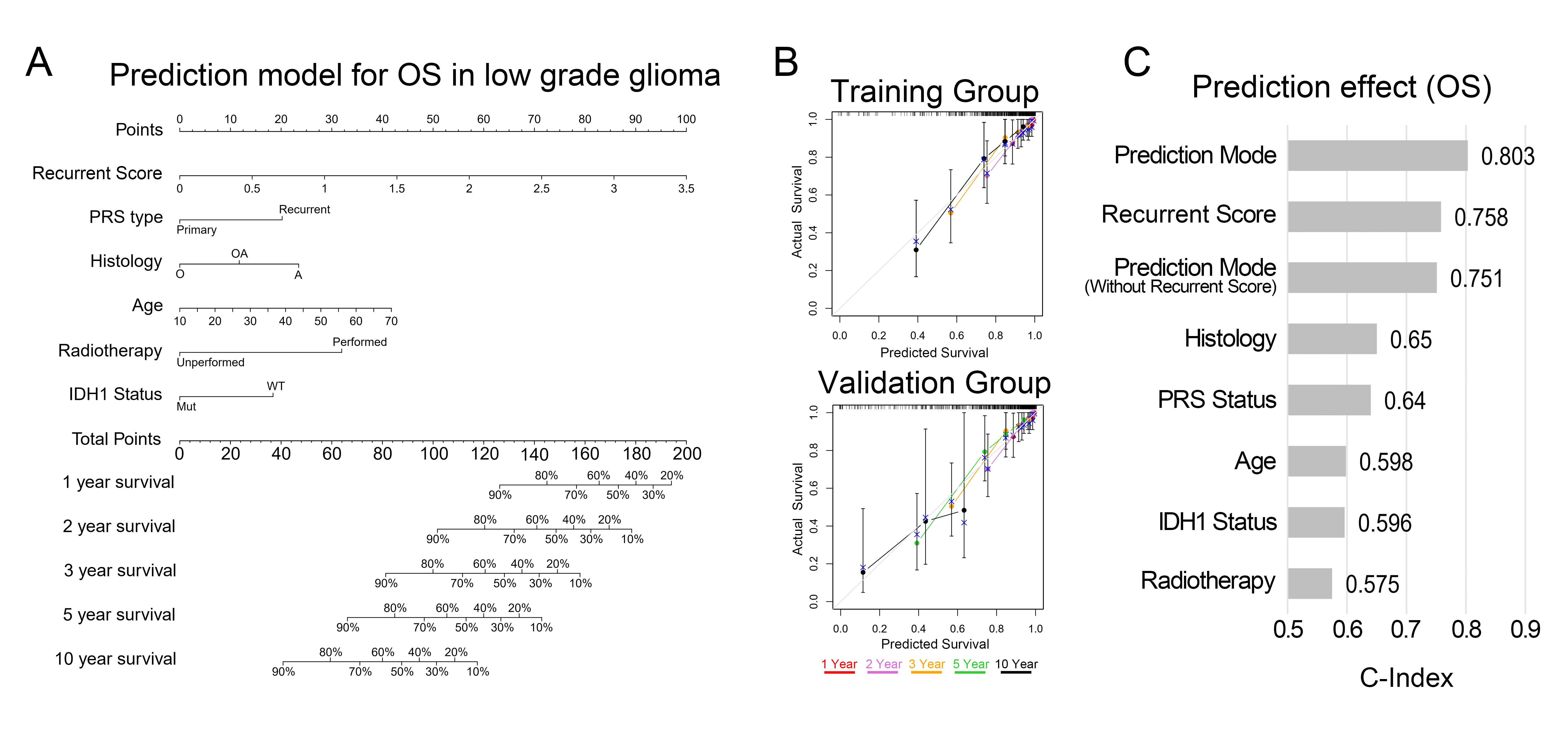

Supplement: Supplementary file 2 — Fig S2 [file CNS-27-186-s002.tif]

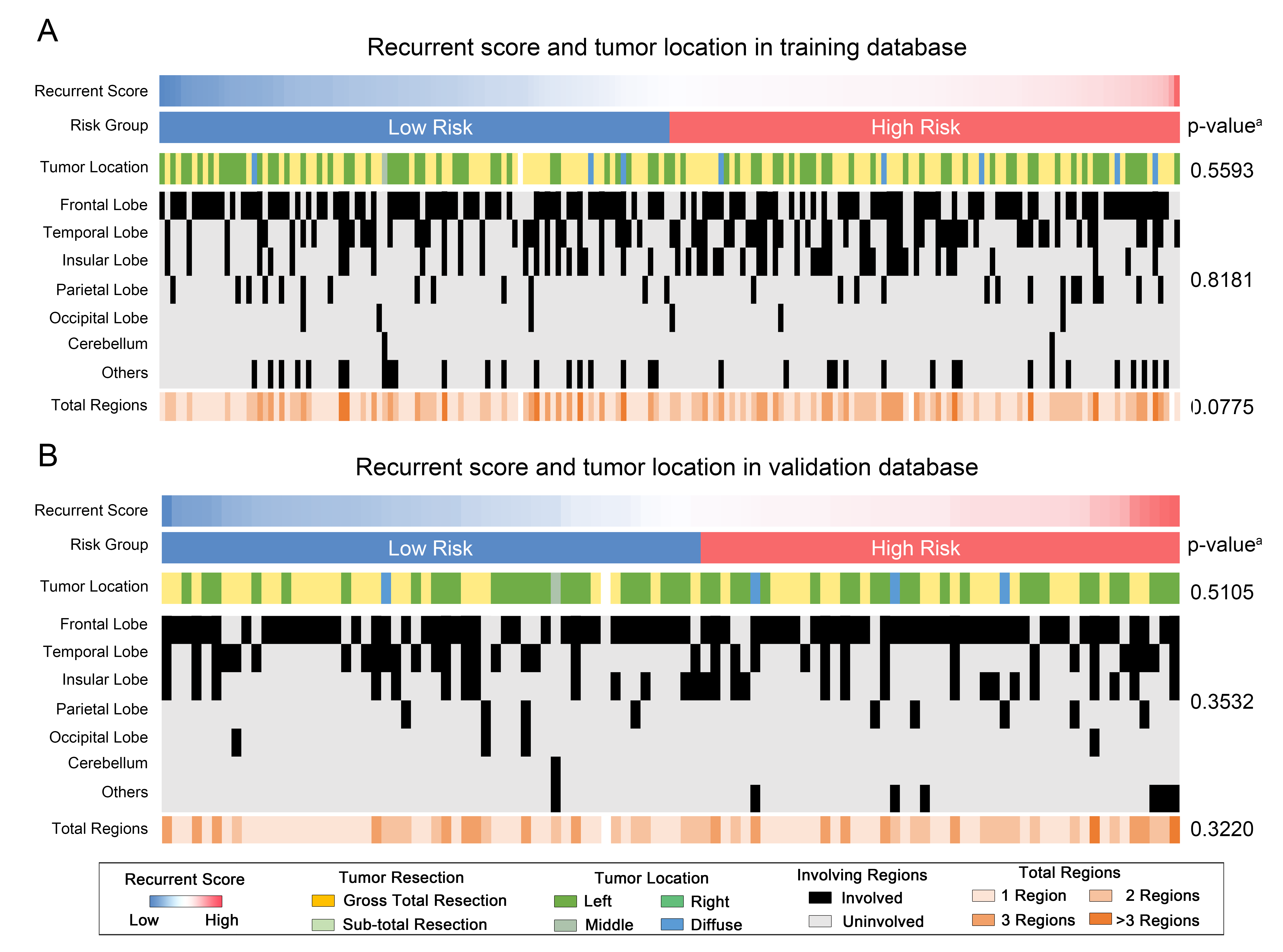

Supplement: Supplementary file 3 — Fig S3 [file CNS-27-186-s003.tif]
