# Supplementary material for: A novel DNA repair‐related nomogram predicts survival in low‐grade gliomas
Source: CNS Neurosci Ther. 2020 Oct 16;27(2):186–95. doi: 10.1111/cns.13464 (PMC7816205; doi:10.1111/cns.13464)
Supplement: Supplementary file 4 — Table S1 [file CNS-27-186-s004.docx]

| **Characteristics** | **No. of Patients (Training)** | **No. of Patients (Validation)** |
| --- | --- | --- |
| ***Age at diagnosis*** |  |  |
| Mean | 39.1 | 39.4 |
| Standard Deviation | 9.76 | 8.79 |
|  |  |  |
| ***Gender*** |  |  |
| Male | 116 | 61 |
| Female | 72 | 41 |
|  |  |  |
| ***Histology*** |  |  |
| Astrocytoma | 64 | 38 |
| Oligodendroglioma | 30 | 26 |
| Oligoastrocytoma | 94 | 38 |
|  |  |  |
| ***IDH1 Mutation*** |  |  |
| Mutation | 135 | 90 |
| Wildtype | 37 | 12 |
| Not Available | 16 | 0 |
|  |  |  |
| ***1p/19q Codeletion Status*** |  |  |
| Codeletion | 58 | 44 |
| Non-Codeletion | 112 | 58 |
| Not Available | 18 | 0 |
|  |  |  |
| ***P/R status*** |  |  |
| Primary | 138 | 93 |
| Recurrent | 50 | 9 |
|  |  |  |
| ***Radiotherapy*** |  |  |
| Yes | 123 | 86 |
| No | 47 | 13 |
| Not Available | 18 | 3 |
|  |  |  |
| ***Chemotherapy*** |  |  |
| Yes | 87 | 28 |
| No | 76 | 65 |
| Not Available | 25 | 9 |

**Table S1.** Clinical information of patients.

Number of patients enrolled in our study was listed.
