# Supplementary material for: A novel DNA repair‐related nomogram predicts survival in low‐grade gliomas
Source: CNS Neurosci Ther. 2020 Oct 16;27(2):186–95. doi: 10.1111/cns.13464 (PMC7816205; doi:10.1111/cns.13464)
Supplement: Supplementary file 5 — Table S2 [file CNS-27-186-s005.docx]

| **Table S2 Univariate and multivariate analysis of prognostic parameters in training group (OS)** | | | | | | |
| --- | --- | --- | --- | --- | --- | --- |
| **Variable** |  | **Univariate analysis** | |  | **Multivariate analysis** | |
|  |  | **HR (95% CI)** | **p Value** |  | **HR (95% CI)** | **p Value** |
| **Recurrent Score** |  | 4.300  （2.722-6.791） | ＜0.0001 |  | 3.312  （2.013-5.449） | ＜0.0001 |
|  |  |  |  |  |  |  |
| **Age at Diagnosis** |  | 1.030  （0.999-1.062） | 0.053 |  |  |  |
|  |  |  |  |  |  |  |
| **Gender** |  | 1.205  （0.654-2.221） | 0.550 |  |  |  |
|  |  |  |  |  |  |  |
| **Histology** |  | 0.482  (0.303-0.767) | 0.002 |  | 0.615  （0.381-0.993） | 0.047 |
|  |  |  |  |  |  |  |
| **IDH Status** |  | 0.228  (0.158-0.329) | ＜0.0001 |  | 0.582  （0.270-1.258） | 0.169 |
|  |  |  |  |  |  |  |
| **1p/19q Codel** |  | 0.776  (0.399-1.507) | 0.454 |  |  |  |
|  |  |  |  |  |  |  |
| **P/R Status** |  | 2.224  (1.201-4.120) | ＜0.0001 |  | 1.916  （0.994-3.694） | 0.052 |
|  |  |  |  |  |  |  |
| **Radiotherapy** |  | 3.149  (1.239-8.003) | 0.016 |  | 2.683  （0.988-7.290） | 0.053 |
|  |  |  |  |  |  |  |
| **Chemotherapy** |  | 1.378  (0.731-2.599) | 0.321 |  |  |  |
