# Supplementary material for: A novel DNA repair‐related nomogram predicts survival in low‐grade gliomas
Source: CNS Neurosci Ther. 2020 Oct 16;27(2):186–95. doi: 10.1111/cns.13464 (PMC7816205; doi:10.1111/cns.13464)
Supplement: Supplementary file 6 — Table S3 [file CNS-27-186-s006.docx]

| **Table S3 Univariate and multivariate analysis of prognostic parameters in training group (PFS)** | | | | | | |
| --- | --- | --- | --- | --- | --- | --- |
| **Variable** |  | **Univariate analysis** | |  | **Multivariate analysis** | |
|  |  | **HR (95% CI)** | **p Value** |  | **HR (95% CI)** | **p Value** |
| **Recurrent Score** |  | 4.150  （2.750-6.265） | ＜0.0001 |  | 3.402  （2.195-5.274） | ＜0.0001 |
|  |  |  |  |  |  |  |
| **Age at Diagnosis** |  | 1.030  （1.005-1.057） | 0.020 |  | 1.033  （1.003-1.064） | 0.029 |
|  |  |  |  |  |  |  |
| **Gender** |  | 0.842  （0.511-1.388） | 0.500 |  |  |  |
|  |  |  |  |  |  |  |
| **Histology** |  | 0.588  (0.403-0.859) | 0.006 |  | 0.584  （0.402-0.849） | 0.005 |
|  |  |  |  |  |  |  |
| **IDH Status** |  | 0.619  (0.348-1.102) | 0.103 |  |  |  |
|  |  |  |  |  |  |  |
| **1p/19q Codel** |  | 0.784  (0.447-1.375) | 0.396 |  |  |  |
|  |  |  |  |  |  |  |
| **P/R Status** |  | 2.197  (1.287-3.749) | 0.004 |  | 1.712  （0.977-3.001） | 0.060 |
|  |  |  |  |  |  |  |
| **Radiotherapy** |  | 2.685  (1.273-5.666) | 0.009 |  | 1.801  （0.833-3.895） | 0.135 |
|  |  |  |  |  |  |  |
| **Chemotherapy** |  | 1.276  (0.744-2.189) | 0.376 |  |  |  |
