# Supplementary material for: A novel DNA repair‐related nomogram predicts survival in low‐grade gliomas
Source: CNS Neurosci Ther. 2020 Oct 16;27(2):186–95. doi: 10.1111/cns.13464 (PMC7816205; doi:10.1111/cns.13464)
Supplement: Supplementary file 7 — Table S4 [file CNS-27-186-s007.docx]

| **Table S4 Univariate and multivariate analysis of prognostic parameters in validation group (OS)** | | | | | | |
| --- | --- | --- | --- | --- | --- | --- |
| **Variable** |  | **Univariate analysis** | |  | **Multivariate analysis** | |
|  |  | **HR (95% CI)** | **p Value** |  | **HR (95% CI)** | **p Value** |
| **Recurrent Score** |  | 3.552  （1.907-6.614） | ＜0.0001 |  | 4.268  （1.830-9.955） | 0.0001 |
|  |  |  |  |  |  |  |
| **Age at Diagnosis** |  | 1.034  （0.994-1.075） | 0.096 |  |  |  |
|  |  |  |  |  |  |  |
| **Gender** |  | 0.527  （0.267-1.036） | 0.063 |  |  |  |
|  |  |  |  |  |  |  |
| **Histology** |  | 0.404  (0.239-0.681) | 0.001 |  | 0.511  （0.243-1.072） | 0.076 |
|  |  |  |  |  |  |  |
| **IDH Status** |  | 2.252  (0.539-9.399) | 0.266 |  |  |  |
|  |  |  |  |  |  |  |
| **1p/19q Codel** |  | 0.156  (0.060-0.404) | ＜0.0001 |  | 0.595  （0.164-2.157） | 0.430 |
|  |  |  |  |  |  |  |
| **P/R Status** |  | 2.029  (0.711-5.789) | 0.186 |  |  |  |
|  |  |  |  |  |  |  |
| **Radiotherapy** |  | 0.572  (0.221-1.481) | 0.250 |  |  |  |
|  |  |  |  |  |  |  |
| **Chemotherapy** |  | 3.724  (1.850-7.499) | ＜0.0001 |  | 3.131  （1.506-6.510） | 0.002 |
