# Supplementary material for: A novel DNA repair‐related nomogram predicts survival in low‐grade gliomas
Source: CNS Neurosci Ther. 2020 Oct 16;27(2):186–95. doi: 10.1111/cns.13464 (PMC7816205; doi:10.1111/cns.13464)
Supplement: Supplementary file 8 — Table S5 [file CNS-27-186-s008.docx]

| **Table S5 Univariate and multivariate analysis of prognostic parameters in validation group (PFS)** | | | | | | |
| --- | --- | --- | --- | --- | --- | --- |
| **Variable** |  | **Univariate analysis** | |  | **Multivariate analysis** | |
|  |  | **HR (95% CI)** | **p Value** |  | **HR (95% CI)** | **p Value** |
| **Recurrent Score** |  | 2.951  （1.644-5.297） | ＜0.0001 |  | 3.246  （1.487-7.083） | 0.003 |
|  |  |  |  |  |  |  |
| **Age at Diagnosis** |  | 1.059  （1.020-1.098） | 0.003 |  | 1.042  （1.007-1.079） | 0.020 |
|  |  |  |  |  |  |  |
| **Gender** |  | 0.599  （0.319-1.123） | 0.110 |  |  |  |
|  |  |  |  |  |  |  |
| **Histology** |  | 0.353  (0.215-0.581) | ＜0.0001 |  | 0.443  （0.229-0.855） | 0.015 |
|  |  |  |  |  |  |  |
| **IDH Status** |  | 0.788  (0.330-1.881) | 0.591 |  |  |  |
|  |  |  |  |  |  |  |
| **1p/19q Codel** |  | 0.167  (0.069-0.401) | ＜0.0001 |  | 0.791  （0.240-2.601） | 0.699 |
|  |  |  |  |  |  |  |
| **P/R Status** |  | 2.908  (1.030-8.214) | 0.044 |  | 2.683  （0.881-8.170） | 0.082 |
|  |  |  |  |  |  |  |
| **Radiotherapy** |  | 0.455  (0.190-1.088) | 0.077 |  |  |  |
|  |  |  |  |  |  |  |
| **Chemotherapy** |  | 4.329  (2.244-8.350) | ＜0.0001 |  | 3.185  （1.586-6.400） | 0.001 |
